# Socioeconomic variation in the burden of chronic conditions and health care provision – analyzing administrative individual level data from the Basque Country, Spain

**DOI:** 10.1186/1471-2458-13-870

**Published:** 2013-09-22

**Authors:** Juan F Orueta, Arturo García-Álvarez, Edurne Alonso-Morán, Laura Vallejo-Torres, Roberto Nuño-Solinis

**Affiliations:** 1 Oberri (The Basque Institute for Health Innovation), Sondika, Bizkaia, Spain; 2 Centro de Salud de Astrabudua, Osakidetza (The Basque Health Service), Erandio, Bizkaia, Spain; 3 Kronikgune (The International Centre of Excellence in Research into Chronicity), Bilbao, Bizkaia, Spain; 4 Clinical Trials Unit, University College London, London, UK; 5 Departamento de Economía de las Instituciones, Estadística Económica y Econometría, Universidad de la Laguna, Tenerife, Spain; 6 Centro de Investigaciones Biomédicas de Canarias (CIBICAN), Tenerife, Spain

## Abstract

**Background:**

Chronic diseases are posing an increasing challenge to society, with the associated burden falling disproportionally on more deprived individuals and geographical areas. Although the existence of a socioeconomic health gradient is one of the main concerns of health policy across the world, health information systems commonly do not have reliable data to detect and monitor health inequalities and inequities. The objectives of this study were to measure the level of socioeconomic-related inequality in prevalence of chronic diseases and to investigate the extent and direction of inequities in health care provision.

**Methods:**

A dataset linking clinical and administrative information of the entire population living in the Basque Country, Spain (over 2 million individuals) was used to measure the prevalence of 52 chronic conditions and to quantify individual health care costs. We used a concentration-index approach to measure the extent and direction of inequality with respect to the deprivation of the area of residence of each individual.

**Results:**

Most chronic diseases were found to be disproportionally concentrated among individuals living in more deprived areas, but the extent of the imbalance varies by type of disease and sex. Most of the variation in health care utilization was explained by morbidity burden. However, even after accounting for differences in morbidity, pro-poor horizontal inequity was present in specialized outpatient care, emergency department, prescription, and primary health care costs and this fact was more apparent in females than males; inpatient costs exhibited an equitable distribution in both sexes.

**Conclusions:**

Analyses of comprehensive administrative clinical information at the individual level allow the socioeconomic gradient in chronic diseases and health care provision to be measured to a level of detail not possible using other sources. This frequently updated source of information can be exploited to monitor trends and evaluate the impact of policy reforms.

## Background

The existence of a socioeconomic health gradient is one of the main concerns of public sectors in the European Union [1] and other parts of the world [2]. Despite this, commonly, health information systems do not have reliable data to detect health inequalities and inequities in health care, to assess changes in these over time and to monitor the impact of related policies.

In Spain, the organization of the health care system is devolved to the autonomous regions, which has led to the development of 17 separate regional health services. Financed through taxes, regional health services in Spain provide primary and specialized care, free of charge (except for outpatient prescriptions, for which there is copayment) to all residents. Each person has an assigned general practitioner, who acts as a gatekeeper to the rest of the system. Health care services at all levels of care are predominantly public in most regions, and most health professionals are employees with civil servant status. In general, health care services in each region are owned by a public organization, which centrally oversees the regional health service. In the Basque Country, one of the autonomous regions located in the north of Spain, this organization is called Osakidetza (the Basque Health Service) and was created in 1983.

Nevertheless, the existence of a National Health Service covering all the population and almost free at the point of use does not prevent there being social inequalities in health within the system; indeed, these are known to be present in the Basque Country [3] and in Spain as a whole [4], as there are in the rest of the EU [5]. Moreover, there is some evidence that differences in income can lead to dissimilar access given the same level of need [6] and most published studies show a pro-poor inequality in GP utilization, that is, people in deprived groups are more likely to contact primary care services than those with a higher income, [7], while the better-off are more likely to receive specialized care [8]. In relation to this, high priority is given to the reduction in social health inequalities in Spain and specifically in the Basque Country, where existing Health Plans and the current “Strategy for tackling the challenge of chronicity” explicitly sets this as a goal [9].

The focus of the aforementioned strategy is a new framework, in which the phenomenon of chronicity is considered central, due to the implications of chronic health conditions and multimorbidity for both patients, their families and caregivers, and health systems and societies [10]. Under the strategy, a risk stratification project was launched consisting of the building of a huge dataset that links all the clinical and administrative information available (in the domain of the public health administration) for each resident in the Basque Country.

The aim of this study is to exploit this dataset to measure inequalities and inequities in health and health care delivery for the entire population living in the Basque Country. The richness of this dataset and its systemic scope is one of the strengths of this study, because it overcomes the limitations of most survey-based research and other studies using databases that do not provide a comprehensive picture of prevalence and expenditures across a health system, being limited to certain age groups, diseases or record systems (e.g., only those for primary care).

Our aims are *i)* to estimate, from data in clinical and administrative records from the Basque Health Service, the prevalence of chronic diseases in the population, *ii)* to measure the level of socioeconomic-related inequality in these prevalences, and *iii)* to investigate the extent and direction of inequities in health care provision with respect to socioeconomic deprivation in the Basque Country. Finally, we analyze the implications of our findings for health policies in aged societies.

## Methods

Ethics statement: This study is a part of a project that was approved by the Ethics Committee of the Basque Country. We used databases that employ an opaque identifier to ensure patient confidentiality.

### Data

This study utilized the database prepared by the population stratification program (PREST) of the Basque Health Service. This program was launched in 2010 with the aim of classifying all citizens of the autonomous region in terms of their future care needs. To this end, data recorded since 2007 were collected from various sources, for the following variables: diagnoses (primary care, emergency department, and inpatient), prescriptions, procedures and costs of care. A more detailed description is available elsewhere [11].

The study population included every individual who on 31^st^ August 2011 was covered by public health insurance in the Basque Country and who had been covered for at least 6 months in the previous year, regardless of whether or not they had used or had any contact with the Basque Health Service.

The Spanish National Health System provides almost universal (99.5%) coverage to Spanish citizens and foreign nationals within the Spanish national territory [12]. Only 0.5% of the population falls outside this welfare network, this group consisting of high-income non-salaried individuals who are not obliged to join the social security system [13]. Individuals are included in the PREST database as a function of their insurance status and it is not a requirement that they first make contact with the health services; that is, we observed almost all the inhabitants of the Basque Country.

The database includes individual-level information on age, sex, health care utilization by type of service and diagnosis, Adjusted Clinical Group (ACG) categories, a small area of residence indicator based on census tracts, and the general practice at which they are registered.

### Chronic diseases

We analyzed a comprehensive list of chronic diseases as well as the presence of multimorbidity. In the Basque Health Service, in accordance with the policy of the Ministry of Health, Social Services and Equality of Spain, diagnoses on hospital discharge forms, emergency department databases and primary care medical records are coded according to ICD-9-CM [14], while the ATC [15] coding system is used for drugs prescribed by primary care doctors. With this information, residents in the Basque Country are classified annually using ACGs [16], a case-mix system that enables health problems to be identified from diagnoses and prescriptions, as well as to categorize the residents into one of a hundred groups according to their health care needs and its costs.

In the present study, we analyzed a four-year period of data for each of the patients. For this, we adapted a methodology previously applied in the literature [17]: we developed a list of 52 diseases and defined specific criteria for each one to consider it active. A description of this process is included as Additional file 1. The presence of diseases was determined using all available information in the databases of the Basque Health Service for the four-year period, though patients within the database who were followed-up for less than full four years were also included. This yielded to a total of 2,262,698 individuals, of whom 1,832,086 had an uninterrupted record from 1^st^ September 2007 onwards. Out of the remaining individuals, 95,714 had shorter follow-up periods because they were born after September 2007 or for other reasons, such as, moving house, changes in type of insurance or administrative factors.

Multimorbidity was defined, for the purposes of this study, as the coexistence of more than one health problem in the same patient considering the 52 conditions under study.

### Health care provision

We measured health care provision in terms of cost-weighted utilization of health care. Health care use was estimated for a 12-month period (from 1^st^ September 2010 to 31^st^ August 2011). We consider separately the cost of the following types of services: primary care (including visits to physicians and nurses, laboratory test and radiology examinations), specialized outpatient care (visits to specialists, rehabilitation, dialysis, radiotherapy and chemotherapy services), inpatient stays, emergency department attendances, and prescribing.

In the case of prescribing, the cost was calculated directly from primary care prescriptions recorded in the electronic health records. For the other types of use, the number of services for each patient was multiplied by a standardized cost. The costs of hospitalization and outpatient surgery were calculated in relation to their weight in the corresponding Diagnosis-Related Groups (DRGs). Information on some services was not available and these were therefore excluded from the analysis: admission to psychiatric hospitals, home hospitalization and day care services (except the procedures and services listed above), health care transport, prostheses and other equipment provided to patients at home.

### Socioeconomic measure

The deprivation index of the census tract (median population size = 1,200 inhabitants) of residence proposed by the MEDEA project [18] was used as a proxy for individual socioeconomic position. Five indicators were included in this index (baseline year 2001) based on: rates of unemployment; low educational attainment, overall and among young people (16–29 years); manual workers; and temporary workers.

### Inequality measures

We use a concentration index (CI) as our measure of socioeconomic-related inequality [19]. CIs are bivariate measures of inequality, measuring inequality in one variable (in our case health status and health care costs) related to the ranking of another variable (in our case area deprivation). Following Wagstaff, 2002 [20] the formula for the CI of socioeconomic inequality can be written as follows:

(1) $$\text{CI}=1-\left(2*\left(1-R_{i}\right)\right)\sum_{i=1}^{n}\frac{h_{i}}{n\mu }$$

where *n* is the sample size; *h*_*i*_ is the health/health care measure; *μ* is the average health/health care measure; and $R_{i}=\frac{i}{n}$ is the fractional rank in the socioeconomic distribution of the i^th^ person, where *i* =1 for the poorest and *i* = n for the richest. The CI takes a positive (negative) value when there is socioeconomic-related inequality favoring the rich (poor).

One of the main advantages of CIs is that they make it possible to summarize the extent of inequality in a single measure that can be used to compare inequality levels across time, areas or, as in our case, disease groups and types of services.

A problem with CIs of binary variables is that they are mean dependent [21]. To account for the binary nature of the health indicators used in this study, we apply the normalization proposed by Wagstaff, 2005. This allows us to compare the levels of inequality in the burden of the different diseases with varying levels of prevalence. We report the level of socioeconomic inequality for each chronic disease and multimorbidity category, separately for males and females and after standardizing by age.

With respect to the analysis of health care costs, socioeconomic inequalities in health care simply reflect that different individuals receive different amounts of care, but they tell us very little about potential *inequity* as they do not take into account differences in needs between individuals [22]. For instance, finding that poorer individuals consume proportionally more health care would not be interpreted as inequity. Therefore, in order to measure inequity, we need to standardize by the different levels of needs across individuals. To do so, we measured *horizontal inequity* as the difference between the CI of actual health care costs and the CI of need-predicted health care costs [23]. Need-predicted health care costs were derived from the predicted values of an ordinary least-squares regression model of actual health care against a number of need- and non-need-related indicators (while we did not want to standardize for the non-need-related variables, omitting them from the regression would have biased the coefficients of the need-related variables [24]). The effects of the non-need-related variables in the prediction were neutralized by setting them equal to their mean values.

We utilized the variables available in our dataset as need and non-need indicators. Classifying variables as need or non-need variables requires making value judgments about which factors ought to affect use and which factors ought not to. Empirical analyses of equity in health service use have commonly classified socioeconomic variables as non-need indicators, and the common practice involves using data on age, gender and morbidity indicators as need measures. We follow this convention in this study. The indicators of need in our models included: sex, age (in this case specified as whether or not the individual is 65 years old or older in order to measure the effect of being elderly on health care cost) and Adjusted Diagnosis Groups (ADGs), which are a component of the ACG case-mix system. ADGs are 32 categories, specifically designed to aggregate diagnoses into groups with similar severity, duration of condition, and needs for health care treatment [16]. We used the deprivation index as a non-need-related indicator in the regression models. One of the useful features of CIs is that it is possible to measure the contribution of different factors (covariates in the regression model) to the inequality indices [25]. We exploit this property and measure separately the contribution of each of the need- and non-need-related factors to the level of observed socioeconomic-inequality in health care costs.

All CIs were adjusted for clustering at level of the general practice where the individual was registered.

## Results

### Summary statistics

The total population was 2,262,686, of which 50.90% were female. The proportion of children (age <18 years) was 15%, and 20% of individuals were over 65. Table 1 shows the distribution of the population by age group and sex, and the prevalence of the 52 chronic conditions considered in this study. Hypertension was the most common condition, present in 19% of the population, followed by anxiety/stress disorders which were more prevalent among females than males (14% vs. 7%, respectively). Diabetes, degenerative joint disease, dyspepsia, malignancies, low back pain and asthma were among the most common conditions in both sexes. On the other hand, hypothyroidism, osteoporosis and depression were more highly prevalent among females, while the prevalence of chronic obstructive pulmonary disease (COPD), ischemic heart disease, and other chronic heart diseases were considerably higher in males.

**Table 1:** Age profile and prevalence of chronic diseases in the Basque Country population

	**MALE**		**FEMALE**			**MALE**		**FEMALE**	
**Total sample**	1,111,047	49.10%	1,151,639	50.90%	**Rheumatoid arthritis and autoimmune and connective tissue diseases**	8,571	0.77%	14,858	1.29%
**By age groups**					**Gout**	19,072	1.72%	3,719	0.32%
**0-17**	175,435	15.80%	164,105	14.26%	**Chromosomal anomalies or Inherited metabolic disorders**	12,765	1.15%	9,527	0.83%
**18-44**	429,365	38.65%	407,960	35.43%	**Heart failure**	10,151	0.91%	11,057	0.96%
**45-64**	313,689	28.23%	319,435	27.74%	**Diverticular disease of intestine**	8,221	0.74%	11,120	0.97%
**65-74**	98,916	8.90%	110,762	9.62%	**Chronic liver or pancreatic disease**	11,942	1.07%	6,358	0.55%
**75+**	93,642	8.43%	149,377	12.98%	**Schizophrenia, affective psychosis or bipolar disorder**	8,068	0.73%	7,073	0.61%
					**Parkinson’s disease**	5,743	0.52%	6,838	0.59%
**Prevalence of chronic disease**					**Viral Hepatitis**	7,083	0.64%	4,510	0.39%
**Hypertension**	207,478	18.67%	223,753	19.43%	**Paralysis or muscular dystrophy**	5,941	0.53%	5,208	0.45%
**Anxiety & other neurotic, stress related & somatoform disorders**	80,755	7.27%	161,981	14.07%	**Irritable bowel syndrome**	3,298	0.30%	6,298	0.55%
**Diabetes Mellitus**	70,614	6.36%	60,412	5.25%	**Alcohol problems**	7,037	0.63%	1,887	0.16%
**Degenerative joint disease**	25,897	2.33%	60,878	5.29%	**Epilepsy (currently treated)**	4,729	0.43%	4,162	0.36%
**Treated dyspepsia**	33,624	3.03%	53,088	4.61%	**Inflammatory bowel disease**	4,381	0.39%	4,314	0.37%
**Depression**	19,944	1.80%	59,894	5.20%	**Peripheral vascular disease**	6,675	0.60%	1,698	0.15%
**Hypothyroidism**	11,125	1.00%	67,968	5.90%	**Treated constipation**	2,484	0.22%	4,729	0.41%
**Malignancies**	35,543	3.20%	34,429	2.99%	**Disorders of the immune system**	2,754	0.25%	3,980	0.35%
**Low back pain**	23,777	2.14%	41,352	3.59%	**Migraine**	1,055	0.09%	5,120	0.44%
**Asthma (currently treated)**	30,161	2.71%	33,709	2.93%	**Developmental disorder**	3,750	0.34%	2,272	0.20%
**Glaucoma**	24,827	2.23%	33,274	2.89%	**Chronic sinusitis**	2,518	0.23%	3,369	0.29%
**Osteoporosis**	3,019	0.27%	53,283	4.63%	**Hematologic chronic disorders**	2,598	0.23%	3,140	0.27%
**Atrial fibrillation**	27,818	2.50%	22,860	1.98%	**Psoriasis or eczema**	2,944	0.26%	2,060	0.18%
**Emphysema, chronic bronchitis, COPD**	33,653	3.03%	16,643	1.45%	**Bronchiectasis**	2,137	0.19%	2,863	0.25%
**Peripheral neuropathy, neuritis**	15,561	1.40%	32,028	2.78%	**Other psycho-active substance misuse**	3,791	0.34%	855	0.07%
**Ischemic heart disease**	32,402	2.92%	13,680	1.19%	**Attention deficit disorder**	2,555	0.23%	844	0.07%
**Chronic heart disease, others**	26,611	2.40%	18,637	1.62%	**HIV, AIDS**	1,796	0.16%	839	0.07%
**Cerebro-vascular disease**	22,682	2.04%	22,268	1.93%	**Transplant status**	1,364	0.12%	764	0.07%
**Prostatic hypertrophy**	43,403	3.91%	---	---	**Multiple sclerosis**	628	0.06%	1,308	0.11%
**Deafness, hearing loss**	20,264	1.82%	20,518	1.78%	**Anorexia or bulimia**	109	0.01%	1,377	0.12%
**Dementia**	12,308	1.11%	25,141	2.18%					
**Blindness & low vision**	13,864	1.25%	17,118	1.49%	**Multimorbidity (2+ conditions)**	235,617	21.21%	298,640	25.93%
**Chronic kidney disease**	13,449	1.21%	12,242	1.06%					

The average number of chronic diseases per person was 0.97 and the percentage of the population with two or more of such diseases was 23.61%.

### Inequalities in health

**Figure 1:**
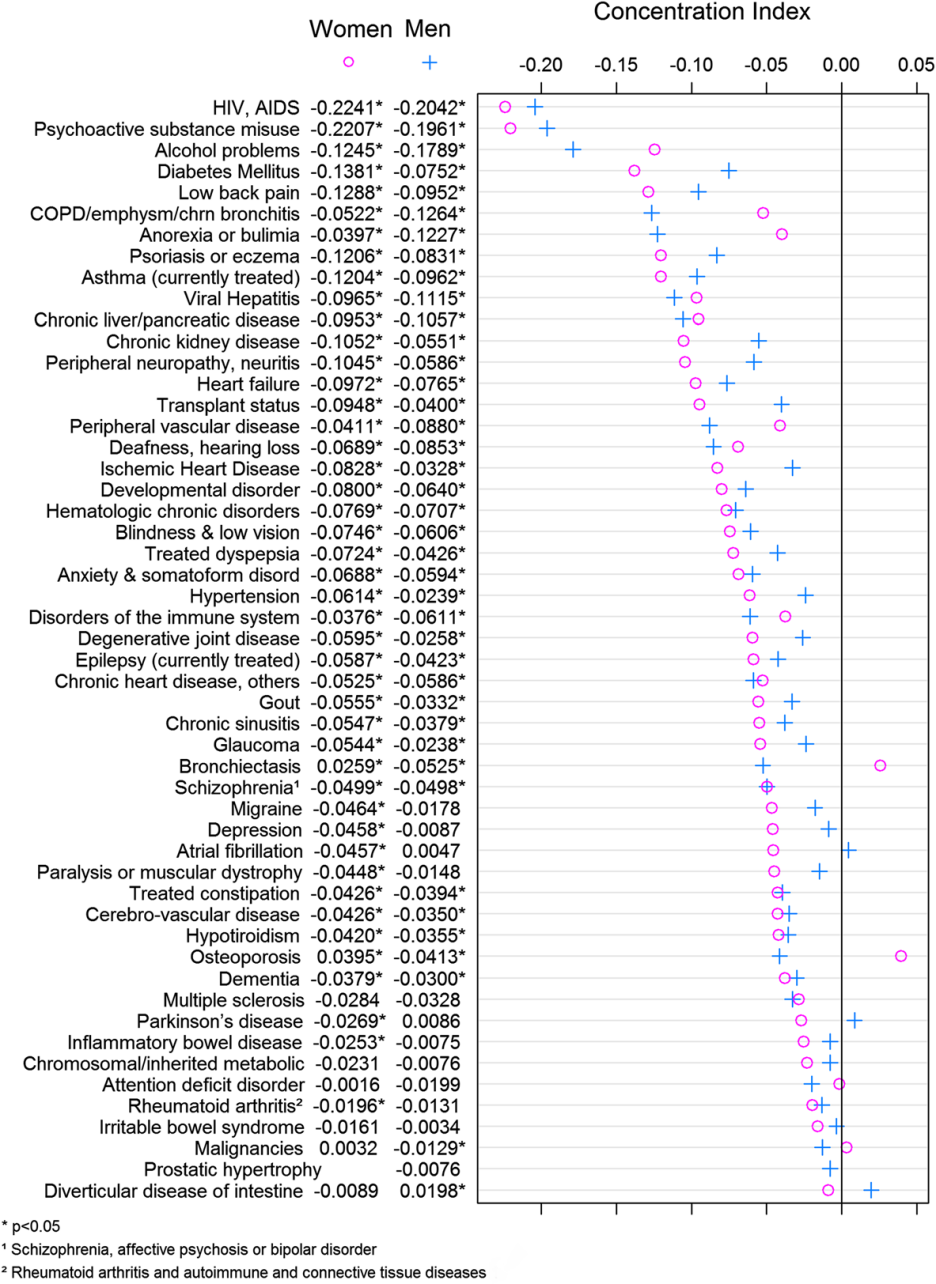
Socioeconomic-related inequality in age-adjusted chronic diseases by sex.

Figure 1 presents the main results with respect to the extent of age-adjusted socioeconomic inequalities in each disease area for males and females. For the vast majority of health conditions, the CIs were found to be negative indicating that the burden of disease is disproportionally concentrated among individuals living in more deprived areas. Interestingly, the steepness of the socioeconomic gradient in the prevalence of diseases varied substantially between diseases, especially among females. HIV, alcohol and drug problems were the health conditions in which we found the greatest socioeconomic inequality among males, while HIV, diabetes and low back pain were the conditions for which inequalities were most pronounced among females. For three different diseases for each sex the concentration index was positive, suggesting that the pathology was disproportionally more prevalent among individuals living in richer areas. These were: atrial fibrillation, Parkinson’s and diverticular disease of the intestine for males (the difference for the last of these being statistically significant); and malignancies, bronchiectasis and osteoporosis for females (differences for the last two statistically significant). For most conditions, the extent of inequality was larger for women than it was for men, with the exception of a few diseases where the gap was notably large: the level of inequality in alcohol problems, COPD, anorexia, and bronchiectasis was much larger among males than females.

**Figure 2:**
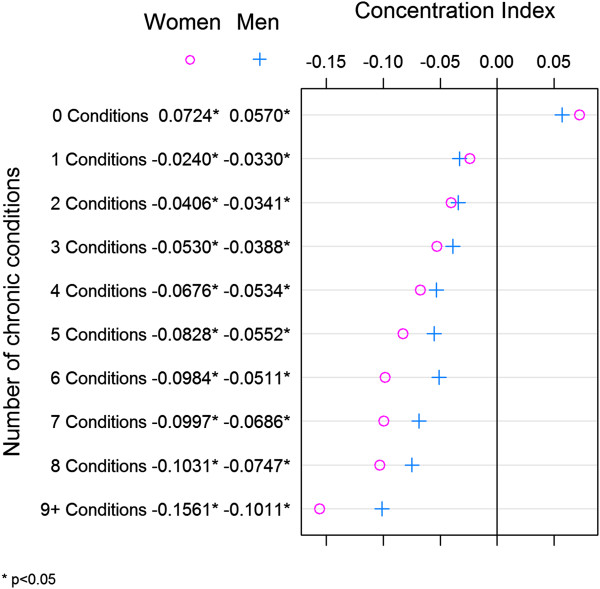
Socioeconomic-related inequality in age-adjusted multimorbidity by sex.

Figure 2 shows the level of socioeconomic inequality in multimorbidity by sex. The results indicate that, after controlling for age, individuals living in more deprived areas had disproportionally more comorbidities than those living in less deprived areas. The extent of inequality observed increased when we defined multimorbidity by the presence of an increasingly larger number of diseases. In every case, there was greater inequality among females than males.

### Inequity in health care provision

Table 2 lists the CIs of observed and need-predicted costs, together with the estimates of horizontal inequity in total health care costs and disaggregated into primary care, specialized outpatient care, emergency department attendances, prescribing and inpatient stays.

**Table 2:** Socioeconomic-related inequity in health care costs

	**Observed CI**		**Need-predicted CI**		**Horizontal inequity**	
	**Female**	**Male**	**Female**	**Male**	**Female**	**Male**
**Primary care**	−0.0655*	−0.0480*	−0.0483*	−0.0391*	−0.0172*	−0.0090*
**Specialized outpatient care**	−0.0764*	−0.0676*	−0.0454*	−0.0417*	−0.0310*	−0.0259*
**Emergency department attendances**	−0.0927*	−0.0720*	−0.0664*	−0.0552*	−0.0263*	−0.0168*
**Inpatient stays**	−0.0844*	−0.0676*	−0.0770*	−0.0752*	−0.0074	0.0077
**Prescriptions**	−0.0662*	−0.0417*	−0.0462*	−0.0361*	−0.0200*	−0.0056
**Total**	−0.0753*	−0.0597*	−0.0569*	−0.0533*	−0.0184*	−0.0065

Observed and need-predicted concentration indices (CIs) and estimates of horizontal inequity in total health care costs.

* p < 0.05.

CIs of observed health care are negative in every case, suggesting that health care provision was disproportionally concentrated among those living in poorer areas for every type of use. After accounting for the differences in the levels of need, the indices of horizontal inequity continued to be pro-poor in most cases, although the magnitude of the indices decreased dramatically and in some cases become non-significant. The values of the indices of horizontal inequity also indicated that the pro-poor bias is greater for specialized outpatient care and emergency department attendance, while the indices were lower for primary care and prescriptions. Among males, inpatient stays appeared to be disproportionally concentrated among richer individuals after accounting for needs, although the horizontal inequity estimate is very small and non-significant. Similar to analysis of inequalities in chronic diseases, we found inequities in health care were also larger among females.

**Figure 3:**
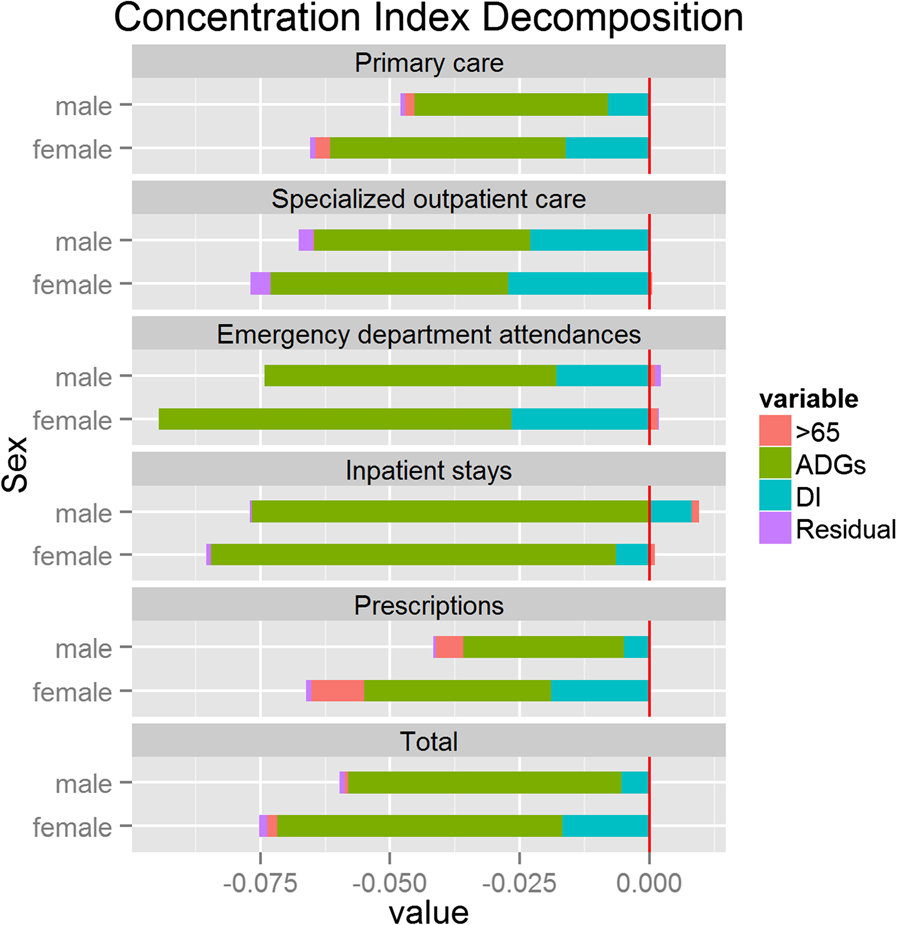
Decomposition of socioeconomic-related inequity in health care cost by type of service and by sex.

The contribution of each factor to explaining the inequalities in observed health care costs are summarized in Figure 3 (note that an alternative and equivalent approach to measuring horizontal inequity is to subtract the contribution of the need-related variables from the inequality index of observed health care). Most of the variation in health care costs was explained by ADG categories. This was followed by the contribution of the deprivation index which, for instance, explained 35% of the pro-poor bias in specialized outpatient care. After controlling for ADG category, the impact of whether an individual was aged 65 or over was relatively small for every type of health care, with the exception of prescriptions where it explained 15 and 13% of the socioeconomic variation in prescription costs in females and males, respectively. Unexplained socioeconomic variation, measured by the contribution of the residual term, was in every case negligible.

## Discussion

The exploitation of administrative health databases in the Basque Country has allowed us to describe the prevalence of chronic diseases, level of multimorbidity and use of health care resources in the entire population and to analyze differences by socioeconomic deprivation and sex. The prevalence of the great majority of the observed chronic diseases is concentrated among the most disadvantaged social groups who also have higher rates of multimorbidity. These inequalities in morbidity burden account for most of the differences found in use of health resources and there is only a small degree of horizontal inequity (difference between use of and need for health care), in most cases this being pro-poor.

Analyzing chronic diseases individually, the largest inequalities are found in pathologies related to high-risk behaviors, substance misuse (smoking, alcohol and other substance abuse) and sedentary life-style, and to exposure to environmental and work-related factors. However, the two sexes do not show analogous patterns in all the conditions and, in general, greater differences were observed in women than men.

Although the methodology used in this study is, in several respects, different to that employed by other authors, our results partially agree with studies in the literature. In a previous study on socioeconomic inequality in the Basque Country, Esnaola et al. found that the greatest differences in causes of mortality between socioeconomic groups were in overdoses of illegal drugs, cirrhosis, AIDS and COPD [3]. Further, a review of data from surveys in eight European countries identified an association between a lower level of education and a higher prevalence of many types of chronic diseases [26], while other research has demonstrated associations between unfavorable socioeconomic conditions and, in particular, cardiovascular disorders [27], diabetes, alcoholism, mental illness, asthma and lumbago, among others. It might seem surprising that, although we found inequalities in the prevalence of stroke (especially in women), these were not as large as the results published by other authors [26]; however, this finding is consistent with a comparative study of causes of mortality across 22 European countries, in which the Basque Country was found to have the lowest levels of inequity associated with level of education for cardiovascular diseases in general, and stroke in particular [28].

On the other hand, for some diseases our results differ from previously published findings. One of the most striking cases is that of skin conditions, which in our study were relatively strongly concentrated in more deprived communities, while other authors have found no relationship between poor socioeconomic conditions and this type of health problem [27,29]. Nevertheless, such an association has been established for certain dermatological conditions [30], so that it seems reasonable to suppose that an analysis separating dermatologic problems into smaller and less heterogeneous groups might have found various types of relationship between social factors and these conditions.

It is well known that the inequalities between social groups in many risk factors vary between the sexes in magnitude and even direction. For example, there are greater inequalities related to level of education for obesity in women, and for smoking [28] and alcohol abuse [31] in men. In relation to this, it seems logical to suppose that social class plays a different role in each sex with regards to the prevalence of specific chronic diseases. These facts may explain, at least to a certain extent, the observed differences in CIs between men and women. In particular, we found that inequity in diabetes is much greater among women, confirming the results of previous studies based on data from surveys and mortality registries [3,26,27,32].

We also found inequity in the prevalence of multimorbidity and this was greater the larger the number of diseases diagnosed in same person. In addition, in all cases, the inequality was larger in women. Although for many years it has been known that the number of people with multiple apparently unconnected conditions is higher than would be expected by chance [33], the interest in studying multimorbidity, as well as its consequences for patients, their families, health care organizations and society in general, is a more recent phenomenon. In recent years, numerous publications have confirmed the relationship between poor socioeconomic status and multimorbidity, using data from primary care medical records [17,34,35], surveys of the general population [36] or users of certain types of health care services [37] and interviews with doctors and patients [38]. Most of these studies have been carried out in developed countries, but there is also some evidence from low and middle-income countries [39].

In the Basque Country, people with poor socioeconomic status use more health resources, especially females. This inequality is mostly explained by the fact that these deprived groups have greater health care needs given their greater morbidity burden. Nevertheless, we still detected a certain degree of pro-poor inequity in the case of specialized outpatient care and emergency services, and to a lesser extent also in primary care and prescriptions, while no inequity was detected in inpatient care. These results are not in agreement with those of other studies that used surveys [8,40-42] or administrative databases [43] as a source of information. These other studies have repeatedly found a pro-rich bias regarding specialized outpatient care, in many different countries in Europe and in the USA. As for primary care, the findings are not so consistent, the direction of the inequity (pro-rich or pro-poor) varying between countries; nevertheless, in general, the differences are smaller and the use of primary care seems to be more related to need than economic factors.

One factor that may partly explain the different use of specialized care observed in our data is that some of the Spanish population, in general those with higher incomes, is covered by private medical insurance as well as the Public Health System [44,45]. Specifically, in 2011, 17.6% of the population of the Basque Country were exclusively covered by private health insurance or had private cover in addition to the public provision [46]. People who are doubly insured often go to private specialized care clinics, in order to avoid the waiting lists for appointments in the Public Health Service. In any case, a previous study carried out in Spain, that included people who have only public health insurance, found pro-rich inequity [47] and, in another one analyzing separately the utilization of public and private health care services, there appeared to be an equitable use of public specialized care, and certainly no pro-poor tendency [48].

Our study has the following strengths. First, it includes almost the entire population of the geographical area studied, thus avoiding selection bias. Second, it exploits a database containing information from primary, specialized outpatient and inpatient care, as well as prescriptions; as other authors have established, the use of a single source can produce inaccurate estimates [49,50], while the complementary use of various sources contributes to a better description of people’s health problems [51]. Furthermore, by cross-checking data on prescriptions and diagnoses it is possible to differentiate between active and non-active chronic diseases and, to achieve this, we adapted a methodology that has already been used by other authors [17]. Thirdly, thanks to this comprehensive data set, our study describes a wide range of specific diseases rather than being limited to self-report general health indicators (which is often the case in survey-based research). It also includes pathologies that are the cause of considerable suffering and disability but not fatal, and so are not considered in studies analyzing only mortality data. Finally, in order to control for morbidity and estimate health care needs we opted to employ the ACG case-mix system, a well-known instrument, the usefulness of which has been confirmed in various different countries.

Our study also has certain limitations. Firstly, administrative databases only contain information about problems for which people seek medical attention. Therefore, the prevalence of diseases can only reflect known cases and excludes the presence of diseases that are present but not known of by the patients or their doctors. The distribution of undiagnosed cases might be influenced by factors including accessibility to health care services and help seeking behavior of patients. Secondly, our database contains information from primary and specialized care, emergency departments and hospital admissions, but not psychiatric hospitals; even though patients admitted in such hospitals are also usually cared for by primary care doctors, given their special characteristics, it is feasible that their health records were not as complete as for the rest of the population. Moreover, as previously noted, we do not have access to information from the health services in the private sector as they are not under the management of the Basque Health Service. As a consequence, in this research we were not able to explore the effect of the existence of double insurance. Thirdly, regarding the cost of care per patient, our health service has no direct data and, hence, costs were calculated from the standard pricing of the services provided and in some specific cases the costs were not available (mainly admissions to mental health hospitals, home hospitalization and some components of day care services). Finally, being based on socioeconomic indicators at the level of area of residence, our study has the limitations common to ecological studies. Although there is known to be a relationship between socioeconomic deprivation and poor health, the contributions of individual and context-related factors have not been clearly established. In any case, the area-based measures, reflecting community-wide characteristics, turn out to be indicators with their own characteristics and do not behave solely as proxy measures for individual socioeconomic variables [52]. The deprivation index employed in this study is a compound index, built from five indicators corresponding to rates of employment and educational attainment [18]. In their first models, the developers of this index included a further nine variables related to housing, sociodemographic characteristics (single parent households, old age, immigrants from low-income countries and recently arrived immigrants) and environmental factors (pollution, noise levels, delinquency). On the basis of the results of multivariate analysis, however, they decided not to include these variables, and to construct the index using the five aforementioned indicators which saturated in the first dimension by the identification of a single factor structure applying principal component analysis. In any case, the information in this index could be insufficiently exhaustive to reflect all the factors of interest.

Although the relationship between low socioeconomic status and poorer health status is complex, and the underlying mechanisms have not been clearly established, the existence of health inequalities is well recognized. Most people in the world aspire to greater solidarity in the field of health and wish their governments to introduce policies that guarantee health protection and promote equity [53]. To face this challenge, it is necessary to develop a broad range of interventions and, as stated by the WHO Commission on Social Determinants of Health [2], we need to develop instruments that measure the magnitude of the problem, facilitate its analysis, and evaluate the effects of said interventions. We need new indicators, different from those currently employed, which only provide average values of health of the general population as a whole [54]. In relation to this, research such as the present study, based on the use of administrative health databases, provides useful information to quantify inequalities and inequities in health and health care provision [55] and, given the accessibility and constant updating of such databases, this approach may help to monitor changes in these inequalities and inequities over time.

## Conclusions

Commonly, health information systems do not have reliable data to detect health inequalities and inequities in health care, to assess changes in these over time and to monitor the impact of related policies. Exploiting a huge dataset that links all the clinical and administrative information available (in the domain of the public health administration) for each resident in the Basque Country, we observed the prevalence of 52 chronic conditions, quantified individual health care costs and estimated the extent and direction of inequality with respect to socioeconomic deprivation. Analyses of comprehensive administrative clinical information at the individual level allow the socioeconomic gradient in chronic diseases and health care provision to be measured to a level of detail not possible using other sources.

## Abbreviations

ACG: Adjusted clinical group; ADG: Adjusted diagnosis group; ATC: Anatomical therapeutic chemical classification system; CI: Concentration index; COPD: Chronic obstructive pulmonary disease; DRG: Diagnosis-related group; EU: European union; ICD-9-C: International classification of diseases, ninth revision, clinical modification.

## Competing interests

The authors declare that they have not competing interests.

## Authors’ contributions

All authors contributed to the design of the study, and participated in the interpretation of data, as well as critically reviewing the manuscript and approving the final version.

## Pre-publication history

The pre-publication history for this paper can be accessed here:

http://www.biomedcentral.com/1471-2458/13/870/prepub

## Supplementary Material

Additional file 1 Definition of chronic conditions. Click here for file
