# Supplementary material for: Socioeconomic variation in the burden of chronic conditions and health care provision – analyzing administrative individual level data from the Basque Country, Spain
Source: BMC Public Health. 2013 Sep 22;13:870. doi: 10.1186/1471-2458-13-870 (PMC3852590; doi:10.1186/1471-2458-13-870)
Supplement: Additional file 1 — Definition of chronic conditions. [file 1471-2458-13-870-S1.docx]

**Additional file**

**DEFINITION OF CHRONIC CONDITIONS**

With the aim of describing the prevalence of chronic diseases and multimorbidity, we adopted a list of 52 pathologies, defined by consensus among the research team. This task was based in an adaptation of work reported in two previous publications by other authors: the 40 diseases selected by Barnett el al. [S1] and the conditions considered as chronic in the ACG Technical Reference Guide [S2].

Barnett and others drew up a list of diseases, based on the impact on patients in terms of need from chronic treatment, impaired function, reduced quality of life and risk of future morbidity or mortality. They included pathologies from a previous systematic review [S3], those included in the Quality and Outcomes Framework (QOF) of the UK General Practice contract and a selection of conditions considered important for health service planning by NHS Scotland. According to the characteristics of each disease, they employed different criteria, which could be: the presence of specific Read codes in the patient medical records, prescription of medications to treat such conditions or a combination of diagnoses and prescriptions. To avoid the presence of inactive health problems, in some situations they established a period for the diagnosis or prescription to be considered.

The ACG system identifies patients suffering specific health problems, by means of codes ICD-9-CM of diagnoses registered or medications prescribed. Based on the clinical characteristics of the condition, diagnoses are also classified into 264 Expanded Diagnosis Clusters (EDCs) and drugs into 69 Rx-defined Morbidity Groups (Rx-MGs). In this case-mix system, a chronic condition is defined as an alteration that is likely to last longer than 12 months and to have a negative impact on health or functional status, and it includes 127 EDCs.

For the purpose of this study, we adapted the list of Barnett et al. to our clinical data, i.e., we identified the conditions from EDCs, Rx-MGs, ICD-9-CM or ATC codes, instead of British National Formulary or Read Codes. We also added to such selection a group of 13 relevant health problems from chronic EDCs. Afterwards, the category “painful condition” was omitted because it contains a very heterogeneous cluster of symptoms and diseases and, therefore, our definitive list was composed of 52 morbidities (Table S1).

S1. Barnett K, Mercer SW, Norbury M, Watt G, Wyke S, et al. (2012) Epidemiology of multimorbidity and implications for health care, research, and medical education: a cross-sectional study. Lancet 380: 37–43. doi:10.1016/S0140-6736(12)60240-2.

S2. Johns Hopkins Bloomberg School of Public Health (2009) The Johns Hopkins ACG Case-Mix System Technical Reference Guide Manual Version 9.0. Baltimore, MD.

S3. Diederichs C, Berger K, Bartels DB (2011) The measurement of multiple chronic diseases--a systematic review on existing multimorbidity indices. J Gerontol A Biol Sci Med Sci 66: 301–311. doi:10.1093/gerona/glq208.

**Table S1: List of chronic morbidities and criteria employed**

| **Chronic condition** | **Criteria** |
| --- | --- |
| Alcohol problems | Dx of Alcohol dependence syndrome (ICD9CM: 303*) ever recorded |
| Anorexia or bulimia | Dx Anorexia or bulimia (ICD9CM=307.1 OR 307.51) ever recorded |
| Anxiety & other neurotic, stress related & somatoform disorders | Dx of Anxiety, neuroses (EDC-PSY01) in the last 12 months OR prescription of anxiolytics OR hypnotics and sedatives (ATC-codes: N05B OR N05C) during 3 or more months |
| Asthma (currently treated) | Dx of Asthma (EDC- ALL04 OR ALL05) ever recorded AND Prescription of drugs to treat Airway Hyperactivity (RxMG-RESx040) in the last 12 months. |
| Atrial fibrillation | Dx of Atrial fibrillation and flutter (ICD9CM: 427.3*) ever recorded |
| Attention deficit disorder | Dx of Attention deficit disorder (EDC-PSY05) ever recorded AND at least 1 prescription of PSYCHOSTIMULANTS, AGENTS USED FOR ADHD AND NOOTROPICS (ATC: N06B) in the last year |
| Blindness & low vision | Dx of Blindness, Retinal disorders, Diabetic retinopathy, Age-related macular degeneration (EDC-EYE02 OR EYE03 OR EYE13 OR EYE15) ever recorded. |
| Bronchiectasis | Dx Bronchiectasis (ICD9CM=494*) ever recorded |
| Cerebro-vascular disease | Dx of Cerebrovascular disease (EDC-NUR05) ever recorded |
| Chromosomal anomalies or Inherited metabolic disorders | Dx of Chromosomal anomalies, Inherited metabolic disorders (EDC-GTC01 OR GTC02) ever recorded |
| Chronic heart disease, others | Dx of Congenital heart disease, Cardiac valve disorders, Cardiomyopathy, Generalized atherosclerosis (EDC-CAR04 OR CAR06 OR CAR07 OR CAR10) ever recorded |
| Chronic kidney disease | Dx of Chronic renal failure, nephritis, nephrosis (EDC-REN01 OR REN04) ever recorded |
| Chronic liver or pancreatic disease | Dx of Chronic liver disease, Chronic pancreatitis (EDC-GAS05 OR GAS12) ever recorded |
| Chronic sinusitis | Dx Chronic sinusitis (ICD9CM=473*) ever recorded |
| Deafness, hearing loss | Dx Deafness, hearing loss (EDC-EAR08) ever recorded |
| Degenerative joint disease | Dx Degenerative joint disease (EDC-MUS03) ever recorded |
| Dementia | Dx Dementia and delirium (EDC-NUR11) ever recorded |
| Depression | Dx of Depression (EDC-PSY09) in the last 12 months OR prescription of Antidepressants (ATC codes N06A ANTIDEPRESSANTS) during at last 4 months in the last year |
| Developmental disorder | Dx Developmental disorder (EDC-NUR19) ever recorded |
| Diabetes Mellitus | Dx of Diabetes Mellitus (EDC-END06 OR END07 OR END08 OR END09) OR Prescription of drugs to treat Diabetes (RxMG-ENDx030 OR ENDx040) ever recorded |
| Disorders of the immune system | Dx Disorders of the immune system (EDC-ALL06) ever recorded |
| Diverticular disease of intestine | Dx Diverticular disease of colon (EDC-GAS10) ever recorded |
| Emphysema, chronic bronchitis, COPD | Dx of Emphysema, chronic bronchitis, COPD (EDC- RES04) ever recorded |
| Epilepsy (currently treated) | Dx of Epilepsy and recurrent seizures (ICD9CM: 345*) ever recorded AND prescription of drugs to treat Seizure Disorder (RxMG-NURx050) in the last year |
| Glaucoma | Dx of Glaucoma (EDC-EYE08) OR prescription of drugs to treat Glaucoma (RxMG-EYEx030) ever recorded |
| Gout | Dx Gout (EDC-RHU02) ever recorded |
| Heart failure | Dx Congestive heart failure (EDC-CAR05) ever recorded |
| Hematologic chronic disorders | Dx of Hemolytic anemia, Hemophilia, coagulation disorder (EDC-HEM01 OR HEM07) ever recorded |
| HIV, AIDS | Dx of HIV, AIDS (EDC-INF04) ever recorded |
| Hypertension | Dx of Hypertension (EDC-CAR14 OR CAR15) OR Prescription of drugs to treat High Blood Pressure (RxMG-CARx030) ever recorded |
| Hypothyroidism | Dx of Hypothyroidism (EDC-END04) OR Prescription of drugs to treat Thyroid Disorders (RxMG-ENDx050) ever recorded |
| Inflammatory bowel disease | Dx Inflammatory bowel disease (EDC-GAS02) ever recorded |
| Irritable bowel syndrome | Dx Irritable bowel syndrome (EDC- GAS09) ever recorded |
| Ischemic Heart Disease | Dx of Ischemic heart disease or acute myocardial infarction (EDC- CAR03 OR CAR12) ever recorded |
| Low back pain | Dx Low back pain (EDC-MUS14) repeated in at least 3 of the last 4 years |
| Malignancies | Dx of Malignacy (MEDC-MAL) ever recorded |
| Migraine | (Dx of Migraine (EDC-NUR22) ever recorded AND prescription of drugs to treat Migraine Headache (RxMG-NURx03) in the last 12 months) OR (more than 4 prescriptions of Antimigraine preparations (ATC: N02C) in the las year) |
| Multiple sclerosis | Dx Multiple sclerosis (EDC-NUR08) ever recorded |
| Osteoporosis | Dx Osteoporosis (EDC-END02) ever recorded |
| Other psycho-active substance misuse | Dx of Drug dependence (ICD9CM: 304*) ever recorded |
| Paralysis or muscular dystrophy | Dx of Muscular dystrophy, Quadriplegia and paraplegia, Spinal cord injury/disorders, Other paralytic syndromes, Cerebral palsy (EDC-NUR09 OR NUR12 OR NUR16 OR NUR17 OR NUR18) ever recorded |
| Parkinson’s disease | Dx of Parkinson's disease (EDC-NUR06) OR Prescription of drugs to treat Parkinson's disease (RxMG-NURx040) ever recorded |
| Peripheral neuropathy, neuritis | Dx Peripheral neuropathy, neuritis (EDC-NUR03) ever recorded |
| Peripheral vascular disease | Dx Peripheral vascular disease (EDC-GSU11) ever recorded |
| Prostatic hypertrophy | Dx Prostatic hypertrophy (EDC-GUR04) ever recorded |
| Psoriasis or eczema | Dx of Psoriasis, dermatitis, eczema (EDC-SKN12 OR SKN02) ever recorded AND prescription during 3 or more months in the last year of “Antipsoriatics” or “Corticosteroids, dermatological preparations Or "Agents for dermatitis, excluding corticosteroids" (ATC: D05 OR DO7 OR D11AH ) |
| Rheumatoid arthritis and autoimmune and connective tissue diseases | Dx of Rheumatoid arthritis or Autoimmune and connective tissue diseases (EDC-RHU05 OR RHU01) ever recorded |
| Schizophrenia, affective psychosis or bipolar disorder | Dx of Schizophrenia and affective psychosis or Bipolar disorder (EDC-PSY07 OR PSY12) ever recorded |
| Transplant status | Dx Transplant status (EDC-ADM03 ) ever recorded |
| Treated constipation | Dx of constipation (EDC-GAS03) ever recorded AND more than 4 prescription of laxatives (ATC: A06) in the last year |
| Treated dyspepsia | Drugs for peptic ulcer and gastro-oesophageal reflux disease (ATC code: A02B) during 3 or more months, without prescription of antiinflammatory and antirheumatic products, non-steroids (ATC code: M01A) NOR Platelet aggregation inhibitors excl. heparin (ATC code: B01AC) in the same month. |
| Viral Hepatitis | Dx Viral hepatitis (ICD9CM=070*) ever recorded |

Dx: Diagnosis

EDC: Expanded Diagnosis Clusters

Rx-MGs: Rx-defined Morbidity Groups.

ICD-9-CM: Spanish version of the Electronic International Classification of Diseases, Ninth Revision, Clinical Modification.

ATC: Anatomical Therapeutic Chemical Classification System
